# Exploring the relationship between menstrual cycle and urticaria: Insights from a cross-sectional study

**DOI:** 10.5415/apallergy.0000000000000206

**Published:** 2025-09-10

**Authors:** Mahdi Shamad, Nahid Osman Ibrahim, Nawaf AlMutairi

**Affiliations:** 1 Faculty of Medicine, Kuwait University, Kuwait; 2 Dermatology Department, College of Medicine, University of Bahri, Sudan; 3 Dermatology Section, Frendship Teaching Hospital, Omdurman, Sudan

**Keywords:** Autoimmune progesterone dermatitis, chronic spontaneous urticarial, cutaneous allergy, menstrual cycle, menstruation, urticaria

## Abstract

**Background:**

Chronic spontaneous urticaria (CSU) is characterized by recurrent wheals lasting over 6 weeks, with various internal and external triggers implicated. Autoimmune progesterone dermatitis, a rare condition associated with cyclical urticaria in response to elevated progesterone levels, may overlap with CSU, particularly in women with menstrual cycle-related exacerbations.

**Objective:**

To document and analyze the timing of urticaria episodes in relation to the menstrual cycle to better understand this phenomenon.

**Methodology:**

This cross-sectional study was conducted at Omdurman Maternity Hospital and Omdurman Friendship Hospital in Sudan from January to June 2022. We included 75 women (ages 18–45) with CSU suspected to be linked to their menstrual cycle. Participants completed a structured questionnaire detailing demographic information, menstrual history, urticaria episodes, and associated symptoms.

**Results:**

Among the 75 participants, 46.7% experienced urticaria exacerbation coinciding with menstruation, primarily within the first 3 days. Another 33.3% reported premenstrual urticaria, while 20% had postmenstrual symptoms. The majority exhibited classic urticarial wheals, with only 3.7% experiencing angioedema. Premenstrual urticaria was significantly associated with the 21 to 30 age group (*P* = 0.030). A majority had a history of allergic conditions, suggesting a predisposition to urticaria influenced by hormonal fluctuations.

**Conclusion:**

This study highlights a significant association between the menstrual cycle and urticaria, suggesting that hormonal fluctuations play a key role in symptom exacerbation. These findings provide a basis for future research into the underlying mechanisms of hormonally influenced urticaria and the development of targeted therapeutic strategies, such as hormonal modulation or individualized treatment plans for affected patients.

## 1. Introduction

Urticaria, commonly referred to as hives, is a common dermatological condition marked by the sudden appearance of wheals—raised, erythematous, and pruritic plaques on the skin [1]. When these symptoms persist for 6 weeks or more without an identifiable external trigger, the condition is classified as chronic spontaneous urticaria (CSU). CSU is characterized by its unpredictable nature, with patients experiencing recurrent outbreaks that can significantly impair quality of life [2]. Despite being termed “spontaneous,” several internal and external factors, such as stress, infections, food additives, and hormonal fluctuations, have been recognized as potential exacerbators of the condition [3–5].

Autoimmune progesterone dermatitis (APD) is a rare, hormonally triggered condition that often manifests with cyclical urticaria, typically in response to the luteal phase of the menstrual cycle, when progesterone levels are elevated [6]. Patients with APD may present with various cutaneous and mucosal symptoms, including eczema, erythema multiforme, aphthous stomatitis, and, most commonly, urticaria [7, 8]. The pathogenesis of APD is not fully understood, but it is thought to involve an autoimmune response to endogenous or exogenous progesterone.

In clinical practice, we have observed a subset of women with CSU who report exacerbations of their symptoms in relation to their menstrual cycle, particularly during the perimenstrual phase. This observation suggests a possible overlap between CSU and hormonally influenced conditions like APD. However, the relationship between the menstrual cycle and urticaria remains underexplored, with limited data available to guide clinical management.

We hypothesized that a subset of women with CSU experience a temporal association between urticaria exacerbation and specific phases of the menstrual cycle, particularly the luteal and early menstrual phases. The aim of this preliminary study was to systematically document and analyze the timing of urticaria episodes in relation to the menstrual cycle among affected women. By establishing a foundational dataset, this study seeks to enhance our understanding of this phenomenon and to provide a basis for further research into the pathophysiology and management of menstrual cycle-related urticaria exacerbations.

## 2. Methodology

This study was designed as a questionnaire-based, cross-sectional, hospital-based investigation aimed at assessing the frequency and characteristics of urticaria related to the menstrual cycle among women attending Omdurman Maternity Hospital and Omdurman Friendship Hospital in Sudan. These hospitals are well-known referral centers for obstetrics and gynecology, and they also have associated dermatology outpatient clinics, making them suitable settings for this study.

### 2.1. Study design and population

The study was conducted over a 6-month period from January to June 2022. Consecutive female patients who presented with CSU and were in the reproductive age group (18–45 years) were included in the study. Patients were selected using a nonprobability convenience sampling method. Inclusion criteria required that participants had a history of urticaria that appeared to correlate with their menstrual cycle and were willing to participate in the study. Patients with chronic urticaria unrelated to their menstrual cycle or those on hormonal therapies were excluded to ensure the specificity of the findings.

### 2.2. Data collection

Data were collected using a structured, predesigned questionnaire developed by the research team based on relevant literature. The questionnaire included sections on demographic details, menstrual history, timing of urticarial episodes in relation to the menstrual cycle, and history of other allergic conditions. The questionnaire also inquired about other variables such as marital status and associated symptoms such as angioedema. The questionnaire was administered by trained nurses in the dermatology outpatient clinics under the supervision of the researchers.

### 2.3. Data analysis

Data were entered and analyzed using SPSS software, version 23. Descriptive statistics were used to summarize the demographic characteristics of the study population and the frequency of urticaria episodes in relation to different phases of the menstrual cycle (premenstrual, menstrual, and postmenstrual). Categorical variables were summarized using frequencies and percentages, while continuous variables were expressed as means ± standard deviations. The chi-square test was employed to assess the association between various categorical factors, with statistical significance set at *P* < 0.05.

### 2.4. Ethical considerations

The study received ethical clearance from the Sudan Medical Specialized Board (chaired by Prof. Aamir A. Hamza, Head of Ethical Committee, Deanship of Academic Research, University of Bahri, Sudan). Written informed consent was obtained from all participating women after thoroughly explaining the study’s nature, purpose, and questionnaire content, emphasizing their right to decline participation at any time. To ensure privacy, patient data was anonymized through coded questionnaires, and the confidentiality of all information was strictly maintained.

## 3. Results

The study included a total of 75 female patients who presented with urticaria associated with their menstrual cycle (50 from Omdurman Maternity Hospital, 25 from Omdurman Friendship Hospital).

### 3.1. Demographic characteristics

The age distribution of the participants ranged from 18 to 45 years with a mean (SD) age of 30.8 (8.8) years. The majority (66.6%) of patients were between 20 and 30 years of age. In terms of marital status, 50 patients (69.4%) were married, 20 patients (26.7%) were single, and 5 patients (6.7%) were divorced. Table 1 summarizes the clinic-demographic characteristics of the study population.

**Table 1.** Demographic, clinical, and allergy characteristics of participants (N = 75)

Parameter	Characteristic	Frequency (n)	Percentage (%)
Age (years)	18–20	8	10.6%
	21–30	50	66.6%
	31–40	9	12%
	41–45	8	10.6%
	Mean (SD)	30.8 (8.8)	
Marital status	Married	50	69.4%
	Single	20	26.7%
	Divorced	5	6.7%
Timing of urticaria episodes	Premenstrual phase	25	33.3%
	Menstrual phase	35	46.7%
	Postmenstrual phase	15	20.0%
Clinical presentation of urticaria	Urticarial wheals	73	97.3%
	Angioedema	2	3.7%
History of allergic conditions	Any allergy	65	86.7%
	Bronchial asthma	10	14.3%

### 3.2. Timing of urticaria in relation to the menstrual cycle

The primary outcome of interest was the timing of urticaria episodes in relation to the menstrual cycle. The study found that the majority of patients (46.7%, n = 35) experienced urticaria during their menstrual cycle, specifically during the first 3 days of menstruation. Another 33.3% of patients (n = 25) reported the onset of urticaria symptoms in the premenstrual phase, typically 3 to 5 days before the onset of menstruation. A smaller proportion of patients (20%, n = 15) experienced urticaria after the menstrual cycle, usually within 2 to 4 days following the cessation of menstruation. A significant correlation was found between premenstrual exacerbation of urticaria and the age group 21 to 30 years (*P* = 0.030, chi-square test, Cramer’s V = 0.117, 95% confidence interval, 0.015–0.214). However, no significant correlation was observed between the timing of urticaria onset and other age groups.

### 3.3. Clinical presentation of urticaria

Regarding the clinical presentation, 97.3% of the patients (n = 73) exhibited classic urticarial wheals characterized by well-demarcated, erythematous, and pruritic plaques on the skin. These wheals were most commonly distributed on the trunk, arms, and thighs, with some patients reporting involvement of the face and neck. Only 2 patients (3.7%) reported experiencing angioedema, which was primarily localized to the lips and eyelids. No patients reported life-threatening angioedema involving the larynx or airway.

### 3.4. History of allergic conditions

All patients in the study had a history of allergic conditions, indicating a possible predisposition to urticaria. Among these, 86.7% (n = 65) reported a history of allergy to a specific substance, such as food allergens, dust, or pet dander. Among these, 14.3% of patients (n = 10) had a documented history of bronchial asthma. No significant correlation was observed between the history of allergic disease and either age group or the timing of urticarial symptom exacerbation.

## 4. Discussion

Our study aimed to investigate the timing of urticaria episodes in relation to the menstrual cycle. We captured data on various types of perimenstrually exacerbated urticaria, including both patients with urticaria exclusively occurring perimenstrually and those with perpetual CSU that exacerbated during menstruation. This approach provided a broader perspective on the interplay between menstrual cycles and urticaria. Patients with urticaria during the premenstrual period are likely to have APD. These different subsets of patients may represent a spectrum within the broader spectrum of urticaria, where hormonal fluctuations significantly contribute to symptom exacerbation. This diversity highlights the complexity of the relationship between hormonal changes and urticaria, underscoring the need to consider both hormonal and nonhormonal factors in the management of CSU.

Our results demonstrate that a significant proportion of patients (46.7%) experienced urticaria during their menstrual cycle, particularly within the first 3 days. Similarly, Ornek et al. [9] noted that 29% of women with chronic urticaria experienced worsening symptoms during the perimenstrual period, which supports the notion that hormonal changes may exacerbate CSU symptoms. Chen et al.‘s study reinforces our study’s finding that urticaria episodes are closely associated with menstrual cycle phases [10]. The study showed that women with abnormal uterine bleeding had a higher risk of developing CSU, suggesting a potential link between menstrual irregularities and chronic urticaria. Case reports have also highlighted the association between CSU and menstruation, including Saul et al. [11], who reported a case of CSU exacerbation premenstrually and noted improvement with suppression of the menstrual cycle.

The pathogenesis of APD and its possible connection to urticaria is of particular interest. Fluctuations in sex hormones, including both progesterone and estrogen, have been implicated in various skin conditions, including urticaria [12]. Our data demonstrated that urticaria was more prevalent before and during the menstrual cycle, with fewer cases occurring postmenstrually. This pattern suggests that APD, characterized by cyclical urticaria in response to elevated progesterone levels, could explain the observed exacerbations, particularly in patients experiencing premenstrual worsening symptoms [13]. Progesterone-induced hypersensitivity may contribute to the exacerbation of CSU symptoms during the luteal phase of the menstrual cycle. The role of these hormones in mast cell degranulation and the pathogenesis of CSU warrants further investigation.

Additionally, the low incidence of angioedema in our study (3.7%) supports findings from Shah et al., who also reported a lower association of angioedema with menstrual cycle-related CSU [14]. Our study found that the majority had a history of allergic conditions, with 86.7% reporting allergies to specific substances like food allergens, dust, or pet dander, suggesting a predisposition to urticaria exacerbations during hormonal fluctuations. The interaction between these allergies and hormonal changes, particularly during the luteal phase of the menstrual cycle, may intensify urticaria symptoms, underscoring the need for an integrated management strategy that addresses both allergenic and hormonal factors [15]. For patients with consistent perimenstrual exacerbations, hormonal modulation therapy—such as menstrual suppression using oral contraceptives or GnRH analogs—may represent a promising avenue for symptom control.

While our findings suggest a temporal association between hormonal fluctuations and urticaria exacerbation, alternative explanations must be considered. Nonsteroidal anti-inflammatory drugs use during menstruation, which was not assessed in this study, could potentially act as a confounding factor and should be explored in future research [16]. Patients on hormonal therapies were however excluded at baseline to reduce the influence of exogenous hormonal modulation.

One of the primary strengths of this study is its focus on a relatively underexplored area—urticaria associated with the menstrual cycle—within a well-defined cohort of reproductive-age women. The use of a questionnaire-based approach allowed for the systematic collection of patient-reported data across 2 major referral hospitals in Sudan, providing valuable preliminary insights into the timing and patterns of urticaria episodes in relation to the menstrual cycle.

However, this study also has several limitations. As a questionnaire-based study, the severity of urticaria could not be measured using standardized tools such as the urticaria activity score (UAS7) or the urticaria control test (UCT). Additionally, the study was conducted in a hospital-based setting using convenience sampling, focusing on patients who sought care, which may not be fully representative of the broader population of women with CSU. As a cross-sectional, observational study based on self-reported data, this research is inherently limited in its ability to establish causality. Rather, it serves as a preliminary exploration of potential associations between the menstrual cycle and urticaria patterns, intended to inform the design of future longitudinal studies.

Further studies are needed to elucidate the relationship between APD presenting as urticaria and CSU with menstrual exacerbation. Future studies should consider a prospective longitudinal design incorporating serial hormonal assays (eg, serum progesterone and estrogen levels) across different menstrual phases. By correlating these hormone levels with validated urticaria severity scores (such as UAS7 or UCT), researchers can assess a potential dose-response relationship between endogenous hormonal fluctuations and symptom exacerbation. Additionally, exploring the role of hormonal therapies in managing CSU patients with perimenstrual exacerbations could provide new therapeutic avenues. Longitudinal studies with larger and more diverse populations would greatly enhance our understanding of this phenomenon.

## 5. Conclusion

Our study reinforces the relationship between the menstrual cycle and urticaria. Although not all cases of urticaria are influenced by hormonal changes, a subset of patients experience worsening symptoms during specific phases of their menstrual cycle. Future research should focus on exploring the underlying hormonal mechanisms and evaluating targeted treatments for hormonally influenced urticaria.

## Acknowledgements

None.

## Conflicts of interest

The authors have no financial conflicts of interest.

## Author Contributions

All authors have read and approved the final version of the manuscript and agree to be accountable for all aspects of the work. The authors confirm that they meet the International Committee of Medical Journal Editors (ICMJE) criteria for authorship.

Mahdi Shamad: Contributed substantially to the conception and design of the study, participated in the acquisition and analysis of data, and was actively involved in drafting and critically revising the manuscript for important intellectual content.

Nahid Osman Ibrahim: Played a key role in data interpretation, contributed to revising the manuscript critically, and provided final approval of the version to be published.

Nawaf AlMutairi: Supervised the overall project, ensured the integrity of the research process from inception to publication, and was involved in all stages of the manuscript, including conceptualization, critical revision, and final approval. All authors agree to be accountable for all aspects of the work and affirm that the manuscript represents honest and original research. Nawaf AlMutairi is the corresponding author.

## References

[1] Kolkhir P Giménez-Arnau AM Kulthanan K Peter J Metz M Maurer M. Urticaria. Nat Rev Dis Primers. 2022;8:61. 36109590 10.1038/s41572-022-00389-z

[2] Keller L Stitt J. Chronic spontaneous urticaria: quality of life and economic impacts. Immunol Allergy Clin North Am. 2024;44:453-467. 38937009 10.1016/j.iac.2024.03.004

[3] Kasperska-Zajac A Brzoza Z Rogala B. Sex hormones and urticaria. J Dermatol Sci. 2008;52:79-86. 18485675 10.1016/j.jdermsci.2008.04.002

[4] Bansal CJ Bansal AS. Stress, pseudoallergens, autoimmunity, infection and inflammation in chronic spontaneous urticaria. Allergy Asthma Clin Immunol. 2019;15:56. 31528163 10.1186/s13223-019-0372-z PMC6737621

[5] Grumach AS Staubach-Renz P Villa RC Diez-Zuluaga S Reese I Lumry WR. Triggers of exacerbation in chronic urticaria and recurrent angioedema-prevalence and relevance. J Allergy Clin Immunol Pract. 2021;9:2160-2168. 34112472 10.1016/j.jaip.2021.04.023

[6] Ljubojević Hadžavdić S Marinović Kulišić S Ljubojević Grgec D Poljanac A Ilić B. Autoimmune progesterone dermatitis diagnosed by lymphocyte transformation test and progesterone provocation test. Acta Dermatovenerol Croat. 2018;26:276-277. 30390735

[7] Aghazadeh N Berry NA Torgerson RR Park MA Davis DMR. Autoimmune progesterone dermatitis: a retrospective case series. Int J Womens Dermatol. 2022;8:e009. 35822192 10.1097/JW9.0000000000000009 PMC9270595

[8] Aghazadeh N Chattha AJ Hartz MF Davis DMR. Autoimmune progesterone dermatitis in the adolescent population. Pediatr Dermatol. 2021;38:380-384. 33368681 10.1111/pde.14423

[9] Ornek SA Suroji Alkilinc A Kiziltac U Kiziltac K Kocaturk E. Effect of puberty, menstruation, pregnancy, lactation, and menopause on chronic urticaria activity. J Cutan Med Surg. 2023;27:466-471. 37537974 10.1177/12034754231191472

[10] Chen TL Yip HT Wang JH Chang CH Huang C Hsu CY Chang C-H. Risk of chronic spontaneous urticaria in reproductive-aged women with abnormal uterine bleeding: a population-based cohort study. J Dermatol. 2021;48:1754-1762. 34462945 10.1111/1346-8138.16109

[11] Wingate-Saul L Rymer J Greaves MW. Chronic urticaria due to autoreactivity to progesterone. Clin Exp Dermatol. 2015;40:644-646. 25754829 10.1111/ced.12632

[12] Raghunath RS Venables ZC Millington GW. The menstrual cycle and the skin. Clin Exp Dermatol. 2015;40:111-115. 25683236 10.1111/ced.12588

[13] Nguyen T Razzaque Ahmed A. Autoimmune progesterone dermatitis: update and insights. Autoimmun Rev. 2016;15:191-197. 26554933 10.1016/j.autrev.2015.11.003

[14] Shah A Resnick D Miller R Canfield S. Chronic urticaria related to menses. J Allergy Clin Immunol. 2011;127:AB102-AB102.

[15] Rakita U Kaundinya T Silverberg JI. Association of atopic dermatitis severity with menstrual worsening of disease in women: a cross-sectional study. J Eur Acad Dermatol Venereol. 2022;36:e197-e199. 34626035 10.1111/jdv.17730

[16] Yeung WYW Park HS. Update on the management of nonsteroidal anti-inflammatory drug hypersensitivity. Yonsei Med J. 2020;61:4-14. 31887794 10.3349/ymj.2020.61.1.4 PMC6938782

